# Neuroprotective Role of Liver Growth Factor “LGF” in an Experimental Model of Cerebellar Ataxia

**DOI:** 10.3390/ijms151019056

**Published:** 2014-10-21

**Authors:** Lucía Calatrava-Ferreras, Rafael Gonzalo-Gobernado, Diana Reimers, Antonio S. Herranz, Adriano Jiménez-Escrig, Juan José Díaz-Gil, María José Casarejos, María Teresa Montero-Vega, Eulalia Bazán

**Affiliations:** 1 Service of Neurobiology, Ramón y Cajal Institute for Health Research (IRYCIS), Madrid 28034, Spain; E-Mails: luciacalatrava@gmail.com (L.C.-F.); rafael.gonzalo@hrc.es (R.G.-G.); diana.reimers@hrc.es (D.R.); antonio.sanchez@hrc.es (A.S.H.); m.jose.casarejos@hrc.es (M.J.C.); 2 Service of Neurology, Ramón y Cajal Hospital, Madrid 28034, Spain; E-Mail: jimeneze@yahoo.com; 3 Service of Experimental Biochemistry, Puerta de Hierro-Majadahonda Hospital, Madrid 28222, Spain; E-Mail: jjdiazgil@yahoo.es; 4 Service of Biochemistry, Ramón y Cajal Institute of Sanitary Research (IRYCIS), Madrid 28034, Spain; E-Mail: teresa.montero@hrc.es

**Keywords:** liver growth factor, neuroregeneration, cerebellar ataxias, neuroprotection, glutamate, GABA

## Abstract

Cerebellar ataxias (CA) comprise a heterogeneous group of neurodegenerative diseases characterized by a lack of motor coordination. They are caused by disturbances in the cerebellum and its associated circuitries, so the major therapeutic goal is to correct cerebellar dysfunction. Neurotrophic factors enhance the survival and differentiation of selected types of neurons. Liver growth factor (LGF) is a hepatic mitogen that shows biological activity in neuroregenerative therapies. We investigate the potential therapeutic activity of LGF in the 3-acetylpiridine (3-AP) rat model of CA. This model of CA consists in the lesion of the inferior olive-induced by 3-AP (40 mg/kg). Ataxic rats were treated with 5 µg/rat LGF or vehicle during 3 weeks, analyzing: (a) motor coordination by using the rota-rod test; and (b) the immunohistochemical and biochemical evolution of several parameters related with the olivo-cerebellar function. Motor coordination improved in 3-AP-lesioned rats that received LGF treatment. LGF up-regulated NeuN and Bcl-2 protein levels in the brainstem, and increased calbindin expression and the number of neurons receiving calbindin-positive projections in the cerebellum. LGF also reduced extracellular glutamate and GABA concentrations and microglia activation in the cerebellum. In view of these results, we propose LGF as a potential therapeutic agent in cerebellar ataxias.

## 1. Introduction

Cerebellar ataxias (CA) include a heterogeneous group of infrequent diseases characterized by lack of motor coordination [1]. According to their etiology, they can be divided into sporadic forms and hereditary diseases. All of them have in common dysfunction of the cerebellum and associated neuronal circuits, in particular spinocerebellar afferents [2,3,4,5,6]. Current therapeutic approaches have assayed the potential activity of intracerebroventricular, peripheral, or intranasal administration of neurotrophic factors such as insulin-like growth factor (IGF-I), or glial-derived growth factor (GDNF), in different experimental models of cerebellar ataxia in rodents [7,8,9,10,11]. Although the above-mentioned treatments yielded promising results, there is not yet an effective therapy for these types of diseases to date [1].

Liver growth factor (LGF) is a hepatic mitogen purified by Díaz-Gil and colleagues some years ago [12]. Following an in-depth chemical and immunological study, they demonstrated that LGF is an albumin-bilirubin complex, the concentration of which is nearly undetectable in sera from healthy humans or rats, but dramatically increases in the presence of hepatobiliary disorders or liver injury [13,14]. Recent studies show that LGF promotes proliferation of different cell types [15,16,17,18,19] and the regeneration of damaged tissues, including brain tissue. Thus, the intracerebral infusion and peripheral administration of LGF stimulates the sprouting of DA terminals in the striatum of hemiparkinsonian rats [20,21], and promotes the expansion of neural precursors and the generation of new neurons in this experimental model of Parkinson’s disease [20]. Moreover, its delivery into the brain enhances cell viability of grafted neural stem cells, and favors their differentiation to an endothelial-like phenotype [22].

Considering the possibility that LGF could be used as a therapeutic agent in CA, we analyzed its potential neuroregenerative and/or neuroprotective activity in the 3-acetylpyridine (3-AP) experimental model of CA in rats. The neurotoxin 3-AP selectively lesions calbindin-expressing neurons in the inferior olive [7], and this nucleus plays a key role in the control of the cerebellar function by sending glutamatergic excitatory signals to Purkinje cells (PC) [23,24]. Here, we report that intraperitoneal administration of LGF (IP-LGF) reduces neuronal loss in the brainstem and prevents the decrease of calbindin protein expression and immunoreactivity promoted by 3-AP in the cerebellum. Besides, IP-LGF ameliorates motor coordination, as analyzed by rota-rod test, in ataxic rats. In this study, we also show that LGF up-regulates the expression of the anti-apoptotic protein Bcl-2 in the brainstem, and reduces to control levels the basal concentration of glutamate, and the expression of OX6, a Class II antigen expressed by activated microglia, in the cerebellum of 3-AP-lesioned rats.

## 2. Results

### 2.1. LGF Ameliorates Motor Coordination in 3-AP-Lesioned Rats

**Figure 1:**
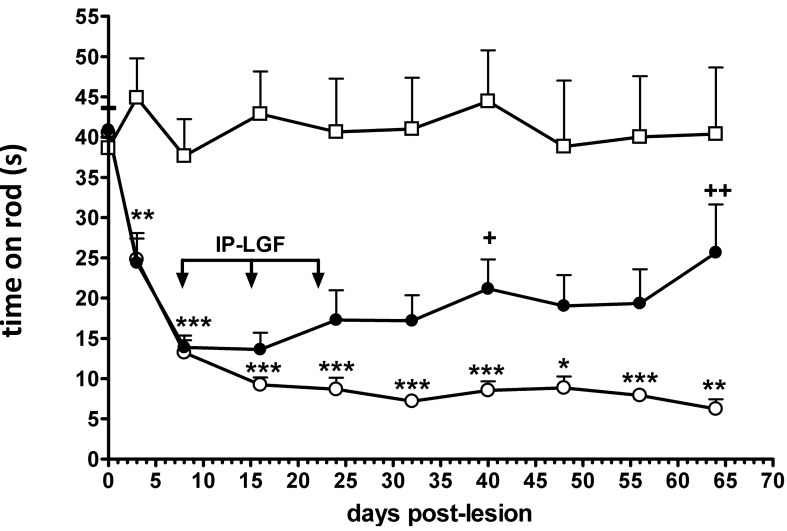
Liver Growth Factor improves motor coordination in 3-acetylpyridine ataxic rats. Motor performance, as assessed by the rotarod test, shows a progressive impairment in 3-acetylpyridine (3-AP)-lesioned rats receiving vehicle that reaches a plateau between 15 and 24 days post-lesion (white circles), as compared with naïve rats (white squares). Note how motor coordination is significantly improved in 3-AP-lesioned rats that received LGF at 40 and 64 days post-lesion (black circles). The latency to fall from rota-rod at 8 days post-lesion was 13 ± 1.6 s (*n* = 15). Results represent the mean ± SEM of 12 to 14 individual animals. * *p* ≤ 0.05, ** *p* ≤ 0.01, *** *p* ≤ 0.001 *vs.* naïve rats, + *p* ≤ 0.05, ++ *p* ≤ 0.01 *vs.* 3-AP + vehicle rats.

To determine whether LGF intraperitoneal (i.p.) administration was functional *in vivo*, we have analyzed motor coordination using the rotarod test. Motor performance of naïve rats was relatively stable over repeated tests while 3-AP-lesioned rats showed a progressive impairment that reached a plateau between 15 and 20 days post-lesion (Figure 1). By contrast, in the 3-AP + LGF group of animals motor performance was stable between 8 and 70 days post-lesion, and significantly improved at 40 and 64 days-post-lesion as compared with 3-AP-lesioned rats receiving vehicle (Figure 1).

### 2.2. LGF Partially Prevents Neurotoxin-Induced Neuronal Loss in the Brainstem and Cerebellum of 3-AP-Lesioned Rats

**Figure 2:**
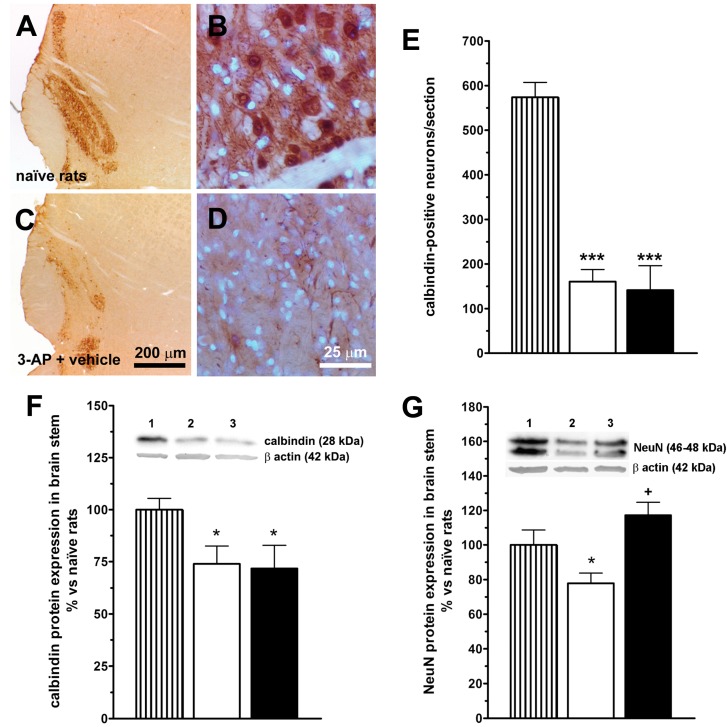
Liver growth factor prevents 3-acetylpyridine-induced neuronal loss in the brainstem. Panels **A** to **D** show calbindin immunostaining in the inferior olive of naïve (**A**,**B**, brown) and 3-acetylpyridine (3-AP) lesioned rats (**C**,**D**, brown); **E** shows the number of calbindin-positive neurons/section in the inferior olive of naïve (**E**, lined bars) and 3-AP-lesioned rats treated with vehicle (**E**, white bars) or LGF (**E**, black bars). Note how 3-AP decreases calbindin-positive cells in this structure; **F** and **G** show the detection by western blot of calbindin (**F**) and neuronal nuclei (**G**, NeuN) in the brainstem. Note how LGF treatment recovers NeuN protein expression in the brainstem of 3-AP-lesioned rats. Lane 1: naïve rats (control); lane 2: 3-AP + vehicle; lane 3: 3-AP + LGF. Results represent the mean ± SEM of 5 to 18 (**E**), or 13 to 18 (**F**), or 4 to 11 (**G**) individual animals. * *p* ≤ 0.05, *** *p* ≤ 0.001 *vs.* naïve rats, + *p* ≤ 0.05 *vs.* 3-AP + vehicle rats. Scale bars: 25 µm (**B**,**D**) and 200 µm (**A**,**C**).

Calbindin is a calcium binding protein expressed by a population of neurons in the inferior olive, and in the cell bodies and terminals of PC in the cerebellum. A single injection of 40 mg/kg 3-AP significantly reduced the number of calbindin-positive neurons in the inferior olive from 574 ± 34 to 161 ± 27 calbindin-positive cells/section in naïve and 3-AP-lesioned rats, respectively (Figure 2A–E). Western blot analysis for calbindin and NeuN, a nuclear antigen expressed by neurons, gave similar results. Thus, in the brainstem of 3-AP + vehicle treated rats calbindin (Figure 2F) and NeuN (Figure 2G) protein levels were significantly lower than those found in naïve rats. LGF treatment did not prevent the reduction in number of calbindin-positive neurons (Figure 2E) and calbindin protein levels (Figure 2F) promoted by the neurotoxin in the inferior olive and brainstem. However, LGF administration up-regulates NeuN protein levels in the brainstem of 3-AP-lesioned rats (Figure 2G).

**Figure 3:**
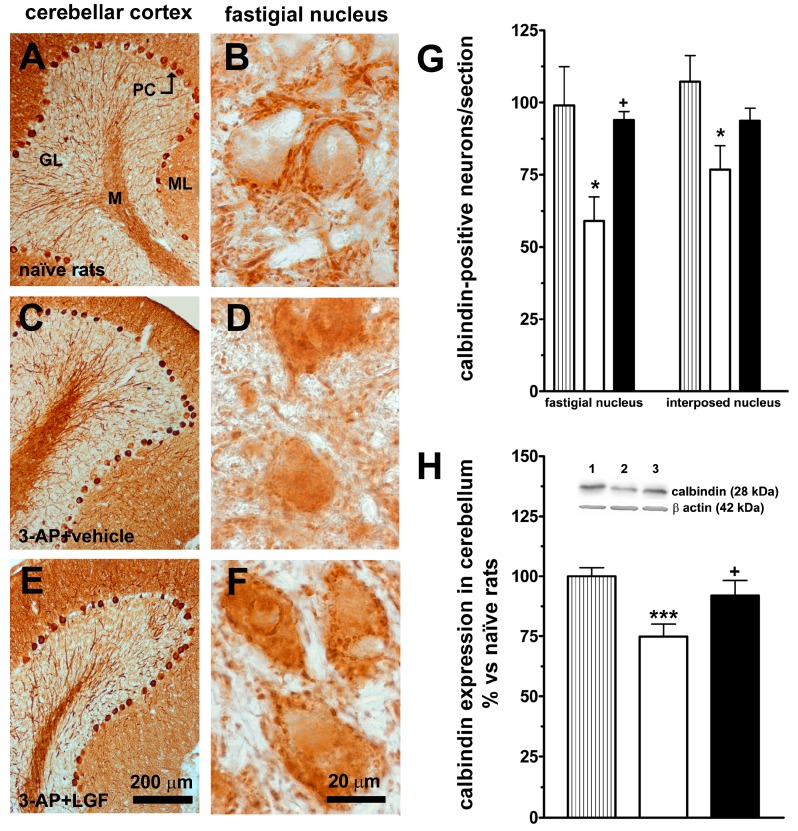
Liver growth factor modulates calbindin protein expression in the cerebellum of 3-acetylpyridine-lesioned rats. **A** to **F** show calbindin immunostaining in the cerebellar cortex (**A**,**C**,**E**, brown) and the fastigial cerebellar deep nucleus (**B**,**D**,**F** brown) of naïve (**A**,**B**), and 3-AP-lesioned rats treated with vehicle (**C**,**D**), or LGF (**E**,**F**); **G** shows the number of neurons that receive calbindin-positive projections in the fastigial and interposed cerebellar deep nuclei and **H** shows the detection by western blot of calbindin in the cerebellum. Note how 3-AP lesion reduces calbindin-positive projections (**G**, white bars) and protein expression (**H**, white bars) in comparison with naïve rats (**G**,**H**, lined bars), and how LGF treatment preserves both parameters (**G**,**H**, black bars). Lane 1: naïve rats (control); lane 2: 3-AP + vehicle; lane 3: 3-AP + LGF. Results represent the mean ± SEM of 4 (**G**) or 12 to 19 (**H**) individual animals. * *p* ≤ 0.05, *** *p* ≤ 0.001 *vs.* naïve rats, + *p* ≤ 0.05 *vs.* 3-AP + vehicle rats. Scale bars: 20 µm (**B**,**D**,**F**) and 200 µm (**A**,**C**,**D**). PC: Purkinje cells, GL: granular layer, ML: molecular layer, M: medulla.

Previously, we reported that 3-AP reduces calbindin protein expression in the cerebellum [25]. Apparently, the neurotoxin did not affect the cell bodies and dendrites of PC in the cerebellar cortex (Figure 3A,C,E), but significantly reduced the number of cells receiving calbindin-positive projections in the fastigial (Figure 3B,D,G) and interposed deep nuclei (Figure 3G). LGF administration preserved calbindin immunoreactivity (Figure 3F), and the number of cells receiving calbindin-positive afferents in both deep nuclei (Figure 3G). Besides, LGF recovered calbindin protein levels to control values in the cerebellum of 3-AP-lesioned rats (Figure 3H).

### 2.3. LGF Regulates Bcl-2 and OX6 Protein Expression in the Brainstem and Cerebellum of 3-AP-Lesioned Rats

**Figure 4:**
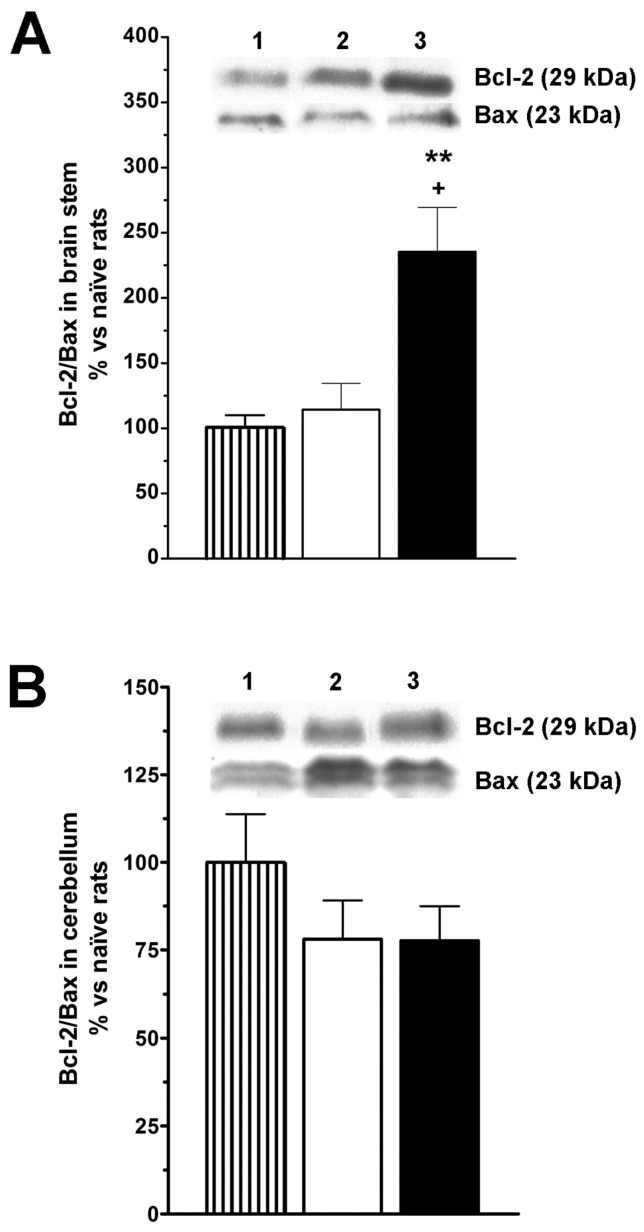
Liver growth factor up-regulates Bcl-2 and protein levels in the brainstem of 3-acetylpyridine ataxic rats. Intraperitoneal administration of LGF raises the ratio Bcl2/Bax in the brainstem of 3-AP-lesioned rats (**A**, black bars) in comparison with naïve (**A**, lined bars) and 3-AP + vehicle treated rats (**A**, white bars). Note how this effect is due to an increase in the expression of the anti-apoptotic protein Bcl-2 (**A**, lane 3), and how LGF treatment does not modulate Bcl2/Bax ratio in the cerebellum (**B**). Lane 1: naïve rats (control); lane 2: 3-AP + vehicle; lane 3: 3-AP + LGF. Results represent the mean ± SEM of 4 to 11 individual animals. ** *p* ≤ 0.01 *vs.* naïve rats, + *p* ≤ 0.05 *vs.* 3-AP + vehicle rats.

In our previous studies we proposed that the neuroprotective activity of LGF is mediated by the regulation of proteins that are critical for cell survival such as Bcl2 [26]. Using western blot analysis, we observed that in the brainstem of 3-AP + LGF treated rats, the ratio Bcl-2/Bax was significantly raised compared to naïve rats and 3-AP + vehicle treated animals (Figure 4A). This effect was due to the increase by 1.3 ± 0.095-fold observed in Bcl-2 protein levels (* *p* ≤ 0.05 *vs.* 3-AP + vehicle rats), while Bax levels remained unchanged in all experimental conditions studied. Independently of the treatment, the cerebellum 3-AP-lesioned rats did not show significant changes in Bcl-2 and Bax protein levels (Figure 4B).

**Figure 5:**
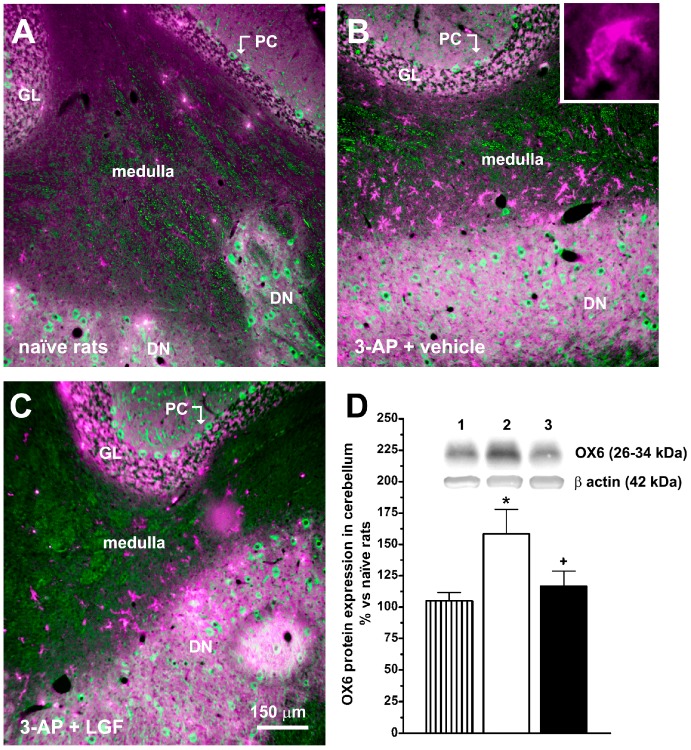
Liver growth factor reduces OX6 immunodetection and protein expression in the cerebellum of 3-acetylpyridine ataxic rats. Panels **A** to **C** show OX6 (magenta) and β-tubulin III (green) immunoreactivity in the cerebellum of 3-AP-lesioned rats. Note that OX6 immunopositive cells in 3-AP-lesioned rats are detected in the medulla near the deep cerebellar nuclei and (**B**) LGF treatment reduces OX6 immunostaining in this structure (**C**) and **D** shows the detection by western blot of OX6 in the cerebellum of naïve rats (**D**, lined bars). Lane 1: naïve rats (control); lane 2: 3-AP + vehicle; lane 3: 3-AP + LGF. Results represent the mean ± SEM of 7 to 15 individual animals. * *p* ≤ 0.05 *vs.* naïve rats, + *p* ≤ 0.05 *vs.* 3-AP + vehicle rats. Scale bar: 150 µm. PC: Purkinje cells, GL: granular layer, DN: deep nuclei.

The neurotoxin 3-AP up-regulates the expression of the histocompatiblility Class II antigen expressed by activated microglia OX6 in the cerebellum [25]. The immunohistochemical analysis revealed the presence of OX6-immunopositive cells in the cerebellar medulla of 3-AP + vehicle rats near the deep nuclei (Figure 5A,B). LGF administration reduced OX6 immunoreactivity in this structure (5C), and OX6 protein levels in the cerebellum of 3-AP-lesioned rats (Figure 5D).

On the other hand, LGF treatment was unable to prevent the increase in OX6 and GFAP protein levels induced by 3-AP in the brainstem and cerebellum, respectively (data not shown).

### 2.4. LGF Modulates the Extracellular Concentration of Glutamate and GABA in the Cerebellar Cortex of 3-AP-Lesioned Rats

**Figure 6:**
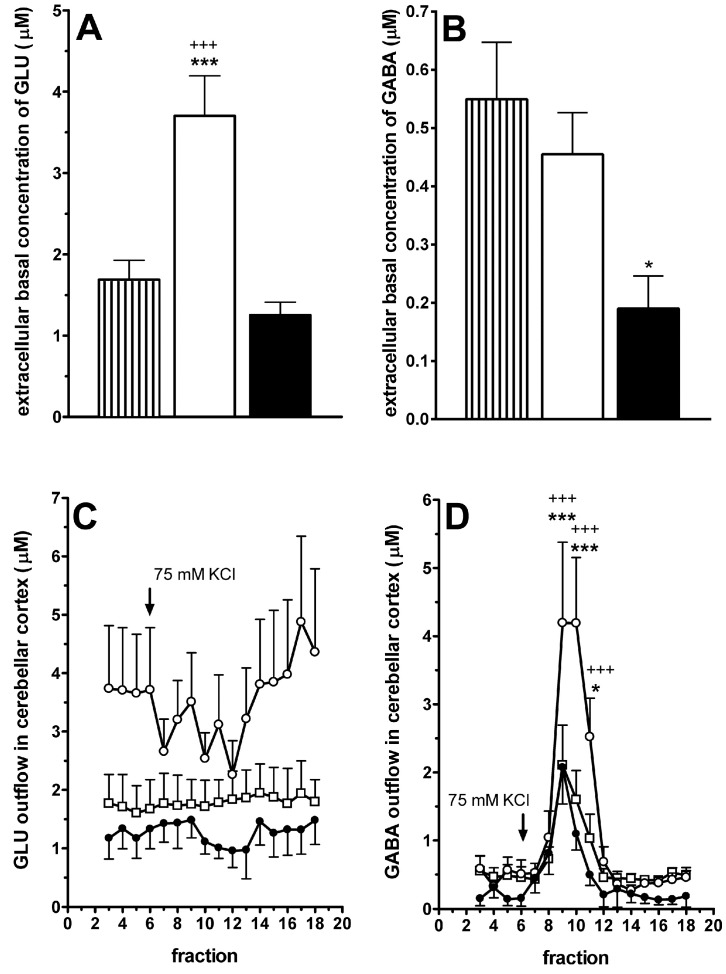
Liver growth factor modulates glutamate and GABA concentration in the cerebellar cortex of 3-acetylpyridine-lesioned rats. Panels **A** and **B** show the extracellular basal concentration of glutamate and GABA in the cerebellar cortex of naïve rats (lined bars), and 3-AP-lesioned rats treated with vehicle (white bars) or LGF (black bars). Note how LGF treatment prevents the increase in the extracellular glutamate basal concentration promoted by 3-AP (**A**), and how the factor also reduces the basal concentration of GABA (**B**); Panels **C** and **D** show the release of glutamate (**C**) and GABA (**D**) in the cerebellar cortex after stimulation with 75 mM KCl. Note how 3-AP increases the release of GABA (**D**, white circles) in comparison with naïve (**D**, white squares) and 3-AP + LGF treated rats (**D**, black circles). Results represent the mean ± SEM of 4 to 8 individual animals. * *p* ≤ 0.05, *** *p* ≤ 0.001 *vs.* naïve rats, +++ *p* ≤ 0.001 *vs.* 3-AP + vehicle rats.

Glutamate and GABA are two neurotransmitters that regulate PC activity levels, which are affected in different types of cerebellar ataxia. As shown in Figure 6A, the extracellular basal concentration of glutamate was significantly increased in the cerebellar cortex of 3-AP + vehicle treated rats as compared with naïve and 3-AP + LGF treated animals. GABA basal concentration was not affected by the lesion with 3-AP (Figure 6B), but after stimulation of neurotransmitters release by KCl, extracellular GABA concentration was significantly higher in the cerebellar cortex of 3-AP-lesioned rats than in naïve animals (Figure 6D). LGF treatment significantly reduced basal GABA concentration below values observed in the cerebellar cortex of naïve rats (Figure 6B). Moreover, LGF administration decreased GABA release to control values (Figure 6D). KCl stimulation did not affect to the extracellular glutamate concentration in the cerebellar cortex of naïve and 3-AP-lesioned rats (Figure 6C), so we were unable to determine if LGF could modulate the release of glutamate in the cerebellum.

## 3. Discussion

In the present study we show that IP-LGF up-regulates the expression of NeuN and Bcl-2 in the brainstem of 3-AP-lesioned rats. LGF treatment also increases calbindin protein expression in the cerebellum, and preserves calbindin-positive projections from 3-AP neurotoxicity in the fastigial and interposed cerebellar deep nuclei. Further, IP-LGF reduces the basal concentration of glutamate and GABA in the cerebellar cortex to control levels, and prevents the activation of microglia promoted by 3-AP in the cerebellum. All these effects were accompanied by a significant improvement in motor coordination, suggesting the potentiality of LGF as a therapeutic agent for cerebellar disorders.

In an experimental model of Parkinson’s disease LGF protects dopaminergic neurons from 6-hydroxydopamine neurotoxicity and ameliorates motor deficits [26]. As we show here, LGF treatment significantly improved motor coordination in ataxic rats, with functional recovery maintained up to one month after the end of the treatment. This beneficial effect could be due to neuronal protection elicited by the factor in the inferior olive, but LGF treatment was unable to preserve calbindin-positive neurons from 3-AP damage in this nucleus of the brainstem. Although LGF did not rescue calbindin-positive neurons from 3-AP toxicity in the inferior olive, our results suggest a potential neuroprotective role of LGF in the brainstem of ataxic rats because IP-LGF administration restored NeuN protein levels to control values, and raised the ratio Bcl2/Bax in this structure of the CNS. This effect was associated with an increase in the expression of Bcl2, a protein involved in cell survival [27,28] that is also up-regulated by LGF in the substantia nigra and striatum of 6-hydroxydopamine-lesioned rats [22,26]. 

Calbindin is a calcium binding protein also expressed by PC in the cerebellum. These cells modulate neuronal activity in the deep cerebellar nuclei that provide the main output from the cerebellum [29]. Here, we show that one single injection of 3-AP significantly reduced calbindin protein expression in the cerebellum, and the number of cells receiving calbindin-positive projections in the fastigial and interposed cerebellar deep nuclei. IP-LGF restored calbindin protein levels in the cerebellum, and preserved calbindin-positive afferents from 3-AP neurotoxicity, suggesting that the behavioral effectiveness of LGF is probably associated with its neuroprotective activity in the cerebellum.

In agreement with a previous study [25], OX6 protein levels were up-regulated in the cerebellum of ataxic rats. Here, we also show that OX6 immunopositive cells were detected in the cerebellar white matter nearby the deep nuclei where calbindin-positive terminals are affected by the neurotoxin. According to our results, LGF could protect the cerebellum by preventing the activation of microglia because its chronic administration reduced OX6 protein expression and OX6 immunoreactivity. Besides, activated microglia contributes to neuronal damage through the release of pro-inflamatory cytokines [30,31], and LGF has a demonstrated anti-inflamatory activity *in vivo* [17,26,32].

Overexpression of GFAP is a feature of reactive astrocytes observed in the cerebellum of ataxic rats [33,34] and in patients with progressive ataxia [35]. We also found increased levels of GFAP in the cerebellum of 3-AP-lesioned rats probably due to the neuronal damage promoted by the neurotoxin in this structure, as it has been reported in other pathologies [36,37,38]. IP-LGF administration could not prevent glial reactivity in the cerebellum.

Our results also show that IP-LGF could regulate PC function by modulating glutamate and GABA homeostasis in the cerebellum of ataxic rats. The inferior olive controls the cerebellar function by sending glutamatergic excitatory signals to PC. The lesion of this nucleus affects the intrinsic properties of PC, which suffer instability in their activity [39,40,41,42]. According to our results, 3-AP administration significantly increased the basal concentration of glutamate in the cerebellar cortex. LGF treatment reduced this parameter to control values. Other studies have proposed that the basal extracellular concentration of neurotransmitters is an index of the affinity their transporters in the CNS [43,44], so we may argue that in the cerebellar cortex of ataxic rats LGF modulates glutamate basal levels by increasing its uptake through the up-regulation of glutamate transporters affinity in the remaining neuronal projections from the inferior olive, and/or in glial cells.

In the CNS the excess of glutamate induces the activation of microglia [45] and neuronal damage [46,47]. In view of the results obtained in this study, we hypothesize that LGF preserves calbindin-positive terminals from 3-AP neurotoxicity by reducing the extracellular glutamate concentration, and consequently the activation of microglia in the cerebellum.

Purkinje cells activity is also modulated through an inhibitory GABAergic input by basket and stellate cells, which are found in the cerebellar molecular layer. 3-AP neurotoxicity did not affect basal GABA concentration in the cerebellar cortex. However, the neurotoxin increased GABA release in this structure contributing to the functional degeneration of PC. These cells control the activity of the deep cerebellar nuclei through GABAergic inhibitory synapses. The impairment of this inhibitory input will increase the firing rate of these cerebellar nuclei, which is in correlation with the ataxia and motor deficits observed in different experimental models in rodents where GABA levels are increased in the cerebellum [34,48,49]. In the 3-AP model of ataxia used in this study, LGF treatment reduced GABA release to control values. The administration of the factor also lowered the extracellular basal concentration of GABA below those values observed in control and 3-AP-lesioned rats, indicating a potential increase in the affinity of GABA transporters in the cerebellar cortex of ataxic rats. GABA transporters are involved in maintaining a low extracellular GABA concentration in brain to prevent an excessive tonic activation of GABA receptors [50]. Besides, GABA transporters could reverse GABA release in normal and pathological conditions [51,52].

## 4. Experimental Section

### 4.1. LGF Purification

LGF was purified from serum of 5-week bile duct-ligated rats following a previously reported procedure [16]. LGF was quantitated by HPLC [53] and samples with the highest serum LGF concentrations were selected to proceed with the purification process, which involved three chromatography steps employing Sephadex G-150, DEAE-cellulose and hydroxylapatite. Purity, that is, the absence of other growth factors and/or contaminants in the LGF preparation, was also assessed according to standard criteria [12,13,14,16,53]. All LGF preparations showed a single band in sodium dodecyl sulfate polyacrylamide gel electrophoresis (SDS-PAGE). LGF preparations were lyophilized and kept at 4 °C until use, at which time aliquots were dissolved in saline for intraperitonal injection.

### 4.2. Ethics Statement

All procedures used in this work were in accordance with the European Union Council Directive (2010/63/EU). The protocol was approved by the Committee on the Ethics of Animal Experiments of the Hospital “Ramón y Cajal” (animal facilities ES280790002001).

### 4.3. Experimental Model of Cerebellar Ataxia in Rats

A total of 100 female Sprague Dawley rats weighing 220–250 g were used in accordance with the European Union Council Directive (86/609/EEC). Rats received an intraperitoneal (i.p.) injection of the neurotoxin 3-AP (40 mg/kg) that selectively damaged calbindin-expressing neurons in the inferior olive [7]. This nucleus plays a key role in the control of the cerebellar function by sending glutamatergic excitatory signals to Purkinje cells (PC) [23,24]. From a histological point of view, PC and granule neurons of the cerebellar cortex are the most commonly affected population of neurons in CA [3].

### 4.4. Behavioral Testing

Motor performance was analyzed using the rotarod test. Before 3-AP lesions were produced, rats received 9 independent training sessions in the rotarod (PanLab S.L., Mod. LE 8500, Cornellá, Spain), with 4 1-min evaluations at 40 rpm (fixed speed), and 4 1-min evaluations at 4 to 40 rpm (accelerating rod). Those animals that withstood more than 1 min at 40 rpm and at 4 to 40 rpm were selected for 3-AP lesions. Motor coordination was evaluated at 72 h and 8 days post-lesion. Those animals with mean latencies to fall on the accelerating rod of approximately 19 ± 3 s (*n* = 62) were selected for LGF or vehicle administration. Starting 8 days after the 3-AP lesion procedure, animals were monitored once a week until the end of the study period.

### 4.5. LGF Administration

Eight days after 3-AP lesions were produced, rats received 2 weekly IP injections of saline (IP-vehicle) or LGF [5 μg/rat] (IP-LGF) for 3 weeks. This optimal dose of LGF has been used in different model systems using an identical or similar schedule [15,16,18,19]. This group of animals was sacrificed 4 weeks after the last treatment with vehicle or LGF. A second group of rats received 2 weekly injections of vehicle or LGF for one week. These animals were used for microdialysis studies 48 h after the last treatment with vehicle (*n* = 5) or LGF (*n* = 4). A total of 37 naïve rats were also used as controls.

### 4.6. Tissue Processing

At 3, 8, 24 and 72 days post-lesion, the animals were perfused intracardially under deep anesthesia with 50 mL of isotonic saline, followed by 250 mL of 4% paraformaldehyde. Brains were postfixed in the same solution for 24 h at 4 °C, cryoprotected and frozen, before sectioning on a cryostat. For the inferior olive 20 μm thick coronal sections were performed at three different levels, following the coordinates of the stereotaxic atlas of Paxinos and Watson [54]: −11.96 mm from Bregma (level 1), −12.80 mm (level 2) and −13.30 mm (level 3).

For immunohistochemical analysis of the cerebellum, 20 μm thick coronal sections were obtained from three different cerebellar levels: the most anterior (−11.30 to −11.60 mm from Bregma) contained the deep nuclei, and the other two levels (−12.80 and −13.30 mm from Bregma, respectively) corresponded to the posterior cerebellum, where a considerable part of climbing fibers originating in the olivary neurons from the previously described level 2, are distributed.

### 4.7. Antibodies and Immunochemicals

The primary antibodies used in this study were: mouse anti-neuronal nuclei (NeuN, 1:1000; Chemicon International Inc., Temecula, CA, USA), mouse anti-OX6 (1:250; AbD Serotec, Oxford, UK), mouse anti Bcl-2 (1:25; Santa Cruz Biotechnology Inc., Burlingame, CA, USA), rabbit anti-calbindin (1:500; Millipore, Temecula, CA, USA), and rabbit anti-Bax (1:250; Santa Cruz Biotechnology Inc., Burlingame, CA, USA). The secondary antibodies and other immunochemicals used were: biotinylated goat anti-mouse IgG (Zymed Laboratories; South San Francisco, CA, USA), streptavidin-biotin-peroxidase complex (DakoCytomation, Carpintería, CA, USA), diaminobenzidine (DAB) + substrate-chromogen system (both from DakoCytomation, Carpintería, CA, USA), Alexa Fluor-568 goat anti-mouse IgG, and Alexa Fluor-488 goat anti-rabbit IgG (1:400; all from Molecular Probes; Eugene, OR, USA).

### 4.8. Immunohistochemistry and Morphometric Analysis

Tissue sections were treated with sodium acetate 10 mM, pH 6.0, at 95 °C for 4 min, and preincubated with 5% normal goat serum (NGS) in Tris-buffered saline (TBS: 0.15 M NaCl and 0.1 M Tris HCl, pH 7.4)/0.1% Triton X-100 for 30 min. Primary antibodies were applied for 24 h at 4 °C, and most of them were visualized using immunofluorescence procedures. The slides were coverslipped in a medium containing p-phenylenediamine and bisbenzimide (Hoechst 33342; Sigma Aldrich, St Louis, MO, USA) for detection of nuclei. Calbindin detection was performed using a biotin-linked secondary antibody, followed by incubation with an avidin-biotinylated peroxidase complex. Finally, the reaction product was detected with DAB chromogen.

For quantitative estimation of calbindin immunostaining in the inferior olive, measurements were performed in several coronal sections, at previously indicated brainstem level 2 (see tissue processing). The area of the olive was delimited and measured in each section, and the number of cells with calbindin staining was assessed using the 20× objective. Immunohistochemical results were expressed as the number of positive cells/section with the aid of the Computer Assisted Stereology Toolbox (CAST)^®^ grid system (Olympus, Ballerup, Denmark). In the case of the cerebellum, the number of neurons receiving calbindin immunoreactive afferents was determined in coronal sections of the anterior cerebellar level herein analyzed (see tissue processing). In order to visualize the fastigial, interposed and dentate nuclei, panoramic images from stitched widefields of each side of the cerebellum were obtained, using the 10X lens and Nikon NIS elements software (scan large image function, Nikon, Tokyo, Japan), connected to a Nikon ECLIPSE Ti-e microscope (Nikon, Tokyo, Japan), equipped with a Nikon DS-2MV camera. The quantitative estimation was expressed as the number of neurons receiving calbindin-positive terminals/section.

### 4.9. Microdialysis Procedure

A total of 17 Female Sprague Dawley rats weighing 220–250 were anesthetized with 2% isoflurane anesthesia and fixed in a stereotaxic frame (David Kopf Instruments, Tujunga, CA, USA). The skin wounds and pressure points were infiltrated with mepivacaine (2%). Body temperature was maintained at 37 °C with a heating pad and monitored by a rectal probe. The microdialysis probes used were from CMA (model N° 11, Kista, Sweden). Each probe was reused no more than three times. Active area of the probe was implanted in the cerebellum cortex following the coordinates: −11.3 mm caudal to bregma, 0 mm lateral to midline and 2.5 mm bellow the dura [54]. The probes were perfused with a standard KRB/KCl 75 mM KRB solutions at a flow rate of 2 µL/min with a syringe minipump (Bionalytical Systems, West Lafayette, ID, USA). A perfusion period of 90 min without sampling was done to enable the recovery of basal levels of amino acids after microdialysis probe implantation. Afterward, perfusate samples were manually collected every 10 min and stored at −20 °C until amino acid analysis.

### 4.10. Amino Acids Determination

The samples were derivatized with ortho-phthal-dialdehyde (OPA). The fluorescent derivatized amino acids were separated by reversed-phase chromatography on a Micra C18 column (33 × 4.6 mm, particle size 1.5 mm) by gradient elution. The mobile phase comprised of solvent A (0.05 M sodium acetate; pH 5.88) and solvent B (methanol) with a binary gradient (time = 0 min %B 2, time = 0.1 min %B 15, time = 1%B 47, time = 6%B 100, time = 9%B 2), at time = 13 min the column is ready for a new sample injection. Ultra pure water and solvents were filtered through 0.2 µm filters from Millipore (Bedford, MA, USA). The solvent flow rate was adjusted to 0.5 mL/min and the injection volume was 10 µL. Fluorescence detection (Perkin-Elmer LS4, Norwalk, CT, USA) was performed at 240 and 450 nm for excitation and emission wavelengths, respectively. Amino acids were identified by their retention times, and their concentrations were calculated by comparing them to calibrated amino acid external standard solutions (1.5 µM).

### 4.11. Western Blotting Protein Analysis

At 72 days post-lesion, the brainstem and cerebellum of 3-AP-lesioned rats that received vehicle (*n* = 15) or LGF (*n* = 11), were removed and dissected following a previously described methodology [55]. Tissue was homogenized (1:8, *w*/*v*) with homogenization buffer (20 mM Tris-HCl, pH 7.5: 140 mM potassium chloride; 5 mM magnesium acetate; 1 mM dithiothreitol, 2 mM benzamidine, 1 mM EDTA, 2 mM EGTA, 0.5% Triton X-100, 10 µg/mL pepstatin A, 10 µg/mL leupeptin and 10 µg/mL antipain; 20 mM sodium β-glycerophosphate; 20 mM sodium molybdate; 200 mM sodium orthovanadate). Homogenates were centrifuged at 11,000× *g* for 20 min, and proteins were processed for Western blot analysis to determine the relative levels of several proteins. The procedures were performed at 4 °C and samples were kept at −80 °C until use. Aliquots of 30 µg of protein were separated by electrophoresis on 10%–15% SDS-polyacrylamide minigels and transferred to nitrocellulose filters. Membranes were soaked in blocking solution (0.1 M PBS and 5% dry skimmed milk, pH 7.4) and incubated with the following primary antibodies diluted in 0.1 M PBS and 1% dry skimmed milk, pH 7.4: mouse anti-Bcl-2 (1:400; Santa Cruz Biotechnology Inc., Burlingame, CA, USA), rabbit anti-Bax (1:300; Santa Cruz Biotechnology Inc., Burlingame, CA, USA), mouse anti-OX6 (1:1000, AbD Serotec, Oxford, UK), mouse anti-NeuN (1:1000; Chemicon International Inc., Temecula, CA, USA), and rabbit anti-calbindin (1:5000; Millipore, Temecula, CA, USA). After extensive washing in 0.05% PBS-Tween, membranes were incubated with the peroxidase-conjugated or alkaline-phosphatase-conjugated secondary antibodies diluted 1:2000 in blocking solution. The membranes were developed with enhanced chemiluminescence Western blotting, following the manufacturer’s instructions (Amersham, Buckinghamshire, England), and were exposed to hyperfilm. Membranes were also immunolabeled for loading control using mouse anti-β actin (1:5000; Sigma Aldrich, St Louis, MO, USA) and anti-mouse IgG alkaline phosphatase-conjugated (1:3000, Sigma Aldrich, St Louis, MO, USA) and were developed with alkaline phosphatase reagent. The density of stained bands was scanned and quantified with the Image QuantTL software package and the data were normalized in relation to β actin levels.

### 4.12. Data Analysis

Results are expressed as mean ± SEM of (*n*) independent animals. Statistical analyses for immunohistochemical and biochemical studies were performed using one-way ANOVA followed by the Newman-Keuls multiple comparison test. For behavioral studies, a two-way ANOVA followed by Newman-Keuls multiple comparison test was used. Differences were considered significant when *p* ≤ 0.05.

## 5. Conclusions

In summary, our study shows that IP-LGF administration has a neuroprotective effect in the brainstem and cerebellum of 3-AP-lesioned rats. In the cerebellum, LGF modulates glutamate and GABA homeostasis by increasing the affinity of their transporters in this structure of the CNS. As a consequence, glutamate and GABA concentrations could be maintained at levels similar to those observed in naïve rats, which may in part explain the improvement in motor coordination observed in 3-AP-lesioned rats. Besides, LGF treatment could protect calbindin-positive terminals from 3-AP neurotoxicity by reducing the extracellular glutamate concentration and the activation of microglia in the cerebellum. Activated microglia are an important source of oxidative stress that could contribute to the etiopathology of cerebellar ataxias; in view of our results, we propose the use of LGF as a potential therapeutic agent for these diseases.
